# Health Risk Assessment of Cadmium in Brazilian Makeup Products Using a Novel MDSPE-FAAS Method

**DOI:** 10.1021/acsomega.6c04274

**Published:** 2026-07-29

**Authors:** Milena Secco de Souza, Cristiane dos Reis Feliciano, Saulo Alves de Souza, Grazielle Cabral de Lima, Nathália Carvalho Costa, Gabriel Oliveira Araújo Costa, Bruno Alves Rocha, Fernando Barbosa, Mariane Gonçalves Santos

**Affiliations:** † Instrumental Analytical Chemistry Research Group − GPQAI, Institute of Chemistry, 74347 Federal University of Alfenas - Unifal-MG, Alfenas, Minas Gerais 37130-000, Brazil; ‡ Analytical and System Toxicology Laboratory, Department of Clinical Analyses, Toxicology and Food Sciences, School of Pharmaceutical Sciences of Ribeirao Preto, University of Sao Paulo, Ribeirão Preto, São Paulo 14040-903, Brazil

## Abstract

The presence of cadmium (Cd) in cosmetic products is a growing public health concern due to its bioaccumulative nature. This study aimed to develop and validate an efficient and environmentally friendly analytical method for the determination of Cd in makeup and to assess the associated health risks. A magnetic dispersive solid-phase extraction (MDSPE) method using Fe_3_O_4_@TEOS nanoparticles was optimized and validated for the quantification of Cd by Flame Atomic Absorption Spectrometry (FAAS). The method was applied to 29 commercial makeup samples (eyeshadows and blushes), including 13 products intended for children. A health risk assessment was conducted based on the guidelines of the Scientific Committee on Consumer Safety (SCCS, 12th Revision) and the United States Environmental Protection Agency (USEPA), evaluating the systemic exposure dose, hazard quotient (HQ), and lifetime cancer risk (LCR). The method demonstrated good linearity (R^2^ = 0.9959), adequate detection limits (0.0016 mg L^–1^) and quantification limits (0.0053 mg L^–1^). Cadmium was detected in 16 of the 29 samples (55.2%), with concentrations reaching up to 25.72 mg kg^–1^. Noncarcinogenic risks were within acceptable limits (HQ < 1), but carcinogenic risks exceeded acceptable limits of the cancer risk slope factor in all cadmium-positive samples, with children’s products showing a 3.4 times higher risk than adult products. The method proved to be reliable and environmentally friendly for monitoring cadmium in cosmetic matrices. The results highlight a potential carcinogenic concern based on conservative risk modeling from Cd exposure through cosmetics, especially for children, underscoring the need for stricter quality control and more effective regulatory oversight in this vulnerable group.

## Graphical abstract

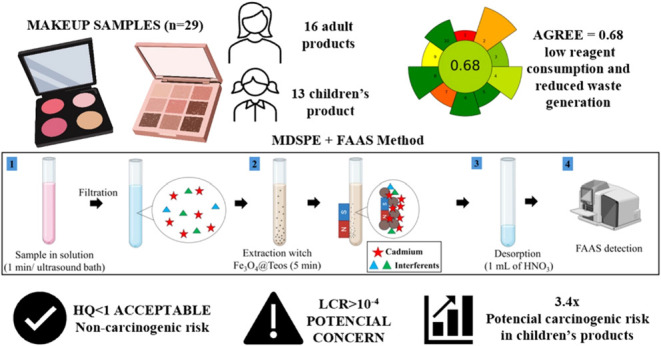

## 1 Introduction

The global use of cosmetic products such as blushes, compact powders, and eyeshadows has increased considerably, raising concerns about the presence of toxic contaminants unintentionally introduced during the manufacturing process.^1,2^ Among these contaminants, toxic metals have attracted significant attention because of their persistence, bioaccumulative behavior, and adverse health effects following chronic exposure. Cosmetic pigments are frequently derived from metallic compounds that may contain impurities of potentially toxic elements.^3^ In particular, cadmium (Cd) is of considerable concern due to its high toxicity, long biological half-life, and tendency to accumulate in human tissues, causing serious toxicological effects after prolonged exposure.^4–6^ Recent reports indicate that Cd contamination in cosmetic products remains a global issue, especially in eye makeup products, highlighting the need for reliable and environmentally friendly analytical methodologies for Cd monitoring in these matrices.^7^ However, the complex composition of cosmetic products still represents a major analytical challenge for trace metal determination.

Conventional sample preparation methods for trace element analysis in cosmetics commonly involve wet digestion using concentrated acids and oxidizing agents or dry ashing at high temperatures.^8^ Although effective, these procedures are often laborious, require specialized instrumentation, generate hazardous chemical waste, and may increase the risk of contamination or analyte loss.^9^ Consequently, the development of faster, simpler, and greener sample preparation strategies remains highly desirable in analytical chemistry.

In this scenario, Magnetic Dispersive Solid-Phase Extraction (MDSPE) has emerged as a promising alternative for trace metal determination because it enables rapid magnetic separation without the need for centrifugation or filtration.^10^ Among the materials employed in MDSPE, Fe_3_O_4_ nanoparticles functionalized with tetraethyl orthosilicate (TEOS) have attracted considerable attention due to their high surface area, low toxicity, and enhanced adsorption properties toward metal ions.^11^ Nevertheless, despite the extensive application of Fe_3_O_4_-based magnetic materials in extraction procedures, their application for Cd determination in cosmetic matrices remains poorly explored. The novelty of the proposed approach lies in combining Fe_3_O_4_@TEOS-based MDSPE with direct FAAS determination for powdered cosmetic samples without conventional acid digestion, enabling a simpler, greener, and low-cost analytical workflow. To the best of our knowledge, no studies have reported the direct application of Fe_3_O_4_@TEOS-based MDSPE coupled with FAAS for Cd determination in powdered cosmetic products without prior acid digestion. In addition, the proposed strategy achieved a lower detection limit (0.0016 mg L^–1^) than several previously reported methods for Cd determination in cosmetics,^12^ which typically present detection limits ranging from 0.004 to 0.02 mg L^–1^, while also reducing reagent consumption and waste generation, reinforcing its alignment with green analytical chemistry principles.^13,14^

Beyond analytical determination, evaluating the potential health risks associated with Cd exposure through cosmetic products is of equal importance. Health risk assessment commonly involves parameters such as systemic exposure dose (SED), hazard quotient (HQ), and lifetime cancer risk (LCR).^15^ Previous studies have reported Cd concentrations in cosmetic products capable of producing unacceptable risk indices, especially for children, who are considered particularly vulnerable to chronic exposure.^16,17^

Accordingly, this work describes the development and validation of a digestion-free and environmentally friendly MDSPE-FAAS method based on Fe_3_O_4_@TEOS magnetic nanoparticles for Cd determination in powdered cosmetic samples. The proposed approach eliminates conventional acid digestion steps and reduces analytical time, reagent consumption, and waste generation. Furthermore, a comprehensive health risk assessment was performed to evaluate the potential noncancerous and carcinogenic risks associated with consumer exposure to Cd-containing cosmetic products.

## 2 Materials and Methods

### 2.1 Chemical and Reagents

Analytical-grade reagents were used in all experimental procedures. TEOS (98%), sodium hydroxide (NaOH, 99%), iron­(II) sulfate tetrahydrate (FeSO_4_·4H_2_O, ≥99%), iron­(III) chloride hexahydrate (FeCl_3_·6H_2_O, ≥98%), and Cd standard solutions (1000 mg L^–1^, ≥99%) were acquired from Sigma-Aldrich (Steinheim, Germany). Ammonium hydroxide (NH_4_OH, 28%) and isopropyl alcohol (99.9%) were obtained from Isofar (Rio de Janeiro, Brazil), while nitric acid (HNO_3_, 65%) and hydrochloric acid (HCl, 37%) were supplied by Exodus Científica (São Paulo, Brazil).

All solvents, including ethanol and deionized water, were of analytical grade. High-purity deionized water (resistivity 18.2 MΩ·cm) was obtained using a Milli-Q water purification system (Millipore RiOs-DI, Bedford, MA, USA) and used for solution preparation, sample dilution, and cleaning procedures. Standard working solutions of Cd were freshly prepared daily by diluting the stock solution with deionized water to the required concentrations. These solutions were stored in amber glass containers at 4 °C until use. All chemicals and solvents were used without further purification.

### 2.2 Synthesis of Fe_3_O_4_@TEOS Nanoparticles

Magnetite nanoparticles were synthesized by the coprecipitation method, following Feliciano et al. (2025).^18^ Briefly, two aqueous solutions containing 3.5 g of FeSO_4_ and 4.0 g of FeCl_3_ in 50.0 mL each were prepared and combined in a three-necked flask under mechanical stirring and a nitrogen atmosphere. A 10.0 mol L^–1^ NaOH solution (25.0 mL) was then added dropwise (0.2 mL min^–1^), and the mixture was stirred for 2 h. The resulting Fe_3_O_4_ nanoparticles were magnetically separated and transferred to a beaker containing 50 mL of ultrapure water. After manual stirring, the solid was again immobilized with an external magnet and the supernatant was removed. The pH of the aqueous supernatant was measured with pH paper, and the washing step (redispersion in water, stirring, magnetic separation, and supernatant removal) was repeated 12 times until the pH of the successive supernatants stabilized near neutrality. Finally, the Fe_3_O_4_ nanoparticles were collected with a magnet and dried in a vacuum oven at 60 °C for 24 h.

The dried Fe_3_O_4_ was sieved and coated with silica using TEOS. For this, 300.0 mg of Fe_3_O_4_ were dispersed in an isopropanol:water mixture (5:1, v/v) by ultrasonic bath for 20 min, followed by the addition of 5.0 mL of 28% NH_4_OH and 2.0 mL of TEOS (98%). The suspension was kept under horizontal stirring at room temperature for 12 h. The Fe_3_O_4_@TEOS nanoparticles were then purified using the same washing sequence described above; however, 24 cycles were required until the pH of the supernatants reached values close to 7.0. The final solid was collected with a magnet and dried in a vacuum oven at 60 °C for 24 h.

### 2.3 Characterization of Magnetic Sorbent

The zeta potential of Fe_3_O_4_@TEOS nanoparticles was characterized using a Zetasizer Nano ZS equipped with an MPT-2 titrator (Malvern, Worcestershire, UK) to evaluate the surface charge and stability of the nanoparticles. Aqueous suspensions (10.00 mL) of Fe_3_O_4_ and Fe_3_O_4_@TEOS (5.00 mg mL^–1^) were prepared individually and subjected to ultrasonic stirring for 30 min to ensure adequate dispersion. Subsequently, 1.00 mL of each suspension was transferred to a 0.02 mol L^–1^ phosphate buffer solution (10.00 mL), and the zeta potential was measured over a pH range of 2.0 to 10.0 to assess the influence of pH on the nanoparticle stability. Morphological and size analyses were performed using a TESCAN MIRA 4 LMU electron microscope with a Schottky emitter (30 kV, 1.2 nm resolution). The analyses were performed in the 5 to 30 keV range.

Powder X-ray diffraction (XRD) analyses were performed using a Rigaku ULTIMA IV automatic diffractometer (Tokyo, Japan) equipped with Bragg–Brentano geometry. Measurements were conducted using Cu Kα radiation at an operating voltage of 40 kV and a current of 30 mA. Scans were performed in continuous mode over a 2θ range of 5 to 70°, with a scan rate of 1.00° min^–1^ and a step size of 0.02°.

Infrared analyses were performed using a Fourier transform infrared (FT-IR) spectrometer (model FT/IR-4X1 Type A, ATR PRO 4X Jasco) with a spectral resolution of 8 cm^–1^. The spectra were recorded in the range of 4000–500 cm^–1^ over 32 scans.

### 2.4 Optimization of Adsorption and Desorption Conditions of Cd^2+^ by Functionalized Nanoparticles

Initially, a preliminary analytical curve was constructed with seven points: 0.025, 0.05, 0.1, 0.3, 0.5, 0.7, and 1.0 mg L^–1^. For the extraction procedure, 25 mg of the sample was weighed in a test tube. Subsequently, 5 mL of the Cd^2+^ standard at pH 6 was added, and the solution was subjected to ultrasonic irradiation for 5 min. Following this step, the sample was filtered, and the filtrate was then added to 10 mg of Fe_3_O_4_@TEOS and agitated at 1000 rpm for 10 min. The magnetic nanoparticles were then isolated using an external magnet, and the solution was discarded. Subsequently, 1 mL of a 0.5 mol L^–1^ HNO_3_ solution was added and agitation was applied for another 5 min. Finally, the magnetic nanoparticles were isolated using a magnet, and the supernatant was recovered, filtered, and analyzed by FAAS.

Following the pilot extraction, the extraction conditions were optimized using a 2^6–1^ fractional factorial design with a central point in triplicate, evaluating six variables: ultrasonic irradiation time, adsorption time, eluent concentration, desorption time, pH, and nanoparticle mass. The coded and actual levels of each factor, as well as the analytical responses, are listed in Table 1, which includes the 32 factorial runs (experiments 1–32) and the three experiments with a central point (experiments 33–35), used to estimate the experimental variance and verify the curvature of the model.

**Table 1:** Factors, Levels, and Analytical Responses Obtained for the 2^6‑1^ Fractional Factorial Design

Experiments	Fe_3_O_4_@TEOS mass (mg)	pH	Eluent concentration (mol L^–1^)	Adsorption time (min.)	Ultrasound time (min.)	Desorption time (min.)	Absorption
**1**	5.00 (−1)	3.0 (−1)	0.100 (−1)	5.0 (−1)	1.0 (−1)	1.0 (−1)	0.052
**2**	20.00 (+1)	3.0 (−1)	0.100 (−1)	5.0 (−1)	1.0 (−1)	10.0 (+1)	0.016
**3**	5.00 (−1)	9.0 (+1)	0.100 (−1)	5.0 (−1)	1.0 (−1)	10.0 (+1)	0.067
**4**	20.00 (+1)	9.0 (+1)	0.100 (−1)	5.0 (−1)	1.0 (−1)	1.0 (−1)	0.171
**5**	5.00 (−1)	3.0 (−1)	1.00 (+1)	5.0 (−1)	1.0 (−1)	10.0 (+1)	0.059
**6**	20.00 (+1)	3.0 (−1)	1.00 (+1)	5.0 (−1)	1.0 (−1)	1.0 (−1)	0.141
**7**	5.00 (−1)	9.0 (+1)	1.00 (+1)	5.0 (−1)	1.0 (−1)	1.0 (−1)	0.103
**8**	20.00 (+1)	9.0 (+1)	1.00 (+1)	5.0 (−1)	1.0 (−1)	10.0 (+1)	0.218
**9**	5.00 (−1)	3.0 (−1)	0.100 (−1)	20.0 (+1)	1.0 (−1)	10.0 (+1)	0.045
**10**	20.00 (+1)	3.0 (−1)	0.100 (−1)	20.0 (+1)	1.0 (−1)	1.0 (−1)	0.102
**11**	5.00 (−1)	9.0 (+1)	0.100 (−1)	20.0 (+1)	1.0 (−1)	1.0 (−1)	0.035
**12**	20.00 (+1)	9.0 (+1)	0.100 (−1)	20.0 (+1)	1.0 (−1)	10.0 (+1)	0.062
**13**	5.00 (−1)	3.0 (−1)	1.00 (+1)	20.0 (+1)	1.0 (−1)	1.0 (−1)	0.053
**14**	20.00 (+1)	3.0 (−1)	1.00 (+1)	20.0 (+1)	1.0 (−1)	10.0 (+1)	0.109
**15**	5.00 (−1)	9.0 (+1)	1.00 (+1)	20.0 (+1)	1.0 (−1)	10.0 (+1)	0.064
**16**	20.00 (+1)	9.0 (+1)	1.00 (+1)	20.0 (+1)	1.0 (−1)	1.0 (−1)	0.539
**17**	5.00 (−1)	3.0 (−1)	0.100 (−1)	5.0 (−1)	10.0 (+1)	10.0 (+1)	0.035
**18**	20.00 (+1)	3.0 (−1)	0.100 (−1)	5.0 (−1)	10.0 (+1)	1.0 (−1)	0.050
**19**	5.00 (−1)	9.0 (+1)	0.100 (−1)	5.0 (−1)	10.0 (+1)	1.0 (−1)	0.061
**20**	20.00 (+1)	9.0 (+1)	0.100 (−1)	5.0 (−1)	10.0 (+1)	10.0 (+1)	0.054
**21**	5.00 (−1)	3.0 (−1)	1.00 (+1)	5.0 (−1)	10.0 (+1)	1.0 (−1)	0.062
**22**	20.00 (+1)	3.0 (−1)	1.00 (+1)	5.0 (−1)	10.0 (+1)	10.0 (+1)	0.077
**23**	5.00 (−1)	9.0 (+1)	1.00 (+1)	5.0 (−1)	10.0 (+1)	10.0 (+1)	0.054
**24**	20.00 (+1)	9.0 (+1)	1.00 (+1)	5.0 (−1)	10.0 (+1)	1.0 (−1)	0.277
**25**	5.00 (−1)	3.0 (−1)	0.100 (−1)	20.0 (+1)	10.0 (+1)	1.0 (−1)	0.033
**26**	20.00 (+1)	3.0 (−1)	0.100 (−1)	20.0 (+1)	10.0 (+1)	10.0 (+1)	0.054
**27**	5.00 (−1)	9.0 (+1)	0.100 (−1)	20.0 (+1)	10.0 (+1)	10.0 (+1)	0.212
**28**	20.00 (+1)	9.0 (+1)	0.100 (−1)	20.0 (+1)	10.0 (+1)	1.0 (−1)	0.018
**29**	5.00 (−1)	3.0 (−1)	1.00 (+1)	20.0 (+1)	10.0 (+1)	10.0 (+1)	0.045
**30**	20.00 (+1)	3.0 (−1)	1.00 (+1)	20.0 (+1)	10.0 (+1)	1.0 (−1)	0.074
**31**	5.00 (−1)	9.0 (+1)	1.00 (+1)	20.0 (+1)	10.0 (+1)	1.0 (−1)	0.133
**32**	20.00 (+1)	9.0 (+1)	1.00 (+1)	20.0 (+1)	10.0 (+1)	10.0 (+1)	0.174
**33**	10.00 (0)	6.0 (0)	0.500 (0)	10.0 (0)	5.0 (0)	5.0 (0)	0.028
**34**	10.00 (0)	6.0 (0)	0.500 (0)	10.0 (0)	5.0 (0)	5.0 (0)	0.039
**35**	10.00 (0)	6.0 (0)	0.500 (0)	10.0 (0)	5.0 (0)	5.0 (0)	0.206

A total of 35 experiments were conducted using a cosmetic matrix composed of blush and eyeshadow, with a fixed sample mass of 25 mg. All experiments were performed with Cd^2+^ at 0.50 mg L^–1^, following the same extraction procedure described for the pilot test, varying only the levels of the factors under study. The analytical response was absorbance measured by FAAS, and the data set (including replicates from the central point) was processed in Excel and Statistica for effect estimation and ANOVA.

The pH study was conducted in a univariate manner, covering a pH range of 8 to 10 at 0.5-unit intervals. For this purpose, 25 mL of a Cd solution at a concentration of 0.5 mg L^–1^ was prepared. The extraction conditions were defined according to the optimal results obtained from the fractional factorial design, following the procedure described above. Subsequently, the solutions were analyzed by FAAS.

### 2.5 Figures of Merit

After optimizing the method, validation was performed in accordance with the recommendations of the Brazilian Health Regulatory Agency (ANVISA), Resolution No. 166, dated July 24, 2017.^19^ The following parameters were evaluated: linearity, sensitivity, precision, accuracy, detection limit, and quantification limit.

Linearity and sensitivity were expressed as the linear correlation coefficient (r) and the angular coefficient of the calibration curve, respectively. These parameters were established using the calibration curve (prepared in sextuplicate) of the analyte at seven different concentrations (0.025, 0.050, 0.10, 0.30, 0.50, 0.70, and 1.0 mg L^–1^). Intraday precision was evaluated using six replicates at three concentration levels (0.025, 0.30, and 1.0 mg L^–1^) on the same day. Interday precision was evaluated using six replicates analyzed at three concentration levels (0.025, 0.30, and 1.0 mg L^–1^) on three different days.

Intra- and interday precision results were evaluated using the coefficient of variation (CV%), while intra- and interday accuracy values were expressed as relative standard error (RSE%). The limit of detection (LOD) and the limit of quantification (LOQ) were determined in accordance with the recommendations of the International Union of Pure and Applied Chemistry (IUPAC) and were calculated based on the noise-associated deviation obtained from ten blank measurements and the slope of the analytical curve. The LOQ was defined as the lowest concentration capable of providing acceptable levels of accuracy and precision, while the LOD corresponded to the lowest concentration that could be statistically distinguished from background noise.

### 2.6 Study of the Interfering Ions

The influence of interfering ions was analyzed individually by comparing the analytical signal obtained for Cd^2+^ in solutions doped with interfering ions and the signal obtained for a solution containing only the ion of interest (Cd^2+^). The analyses were performed according to the previously optimized conditions using binary solutions containing Cd^2+^ at a concentration of 0.5 mg L^–1^ and Ca^2+^, Na^+^, K^+^, Mg^2+^, Fe^2+^, Zn^2+^, Cu^2+^, SO_4_^2–^, and HCO_3_^–^, each at a concentration of 1.0 mg L^–1^.^20,21^ Recoveries between 85 and 115% were considered to indicate no interference from the interfering ions.

### 2.7 Evaluation of the Matrix Effect

The presence of matrix effects in the method was assessed by constructing calibration curves for Cd^2+^ using standard solutions in ultrapure water and in the corresponding matrix. Qualitatively, the matrix effect was assessed by comparing the linear regression lines of the respective calibration curves. Quantitatively, the matrix effect was determined according to eq 1:

1 $$\mathrm{ME}\;\left(\%\right)=\frac{S_{\mathrm{matrix}}-S_{\mathrm{water}}}{S_{\mathrm{water}}}\times 100$$

Where *S*_water_ refers to the slope of the calibration curve constructed from Cd^2+^ standards prepared in ultrapure water, and *S*_matrix_ refers to the slope of the calibration curve constructed from Cd^2+^ standards prepared in the matrix mix of eye shadows.

### 2.8 Application in Real Samples

The validated method was applied to the determination of Cd^2+^ in commercial makeup products acquired from local retailers in Brazil. A total of 29 eye makeup samples, including eyeshadows and blushes, were collected and analyzed. The samples were categorized according to their intended use: 16 products were marketed for adult consumers, while 13 products were specifically designed for children.

Each product was available in multiple color variants, and up to 14 different colors were analyzed per sample, including shades of pink, lilac, black, silver, gold, brown, orange, blue, red, yellow, and green. For each product, Cd^2+^ concentrations were determined individually for each color variant to assess the potential variability in the metal content associated with different pigments.

Sample preparation followed the optimized MDSPE procedure described in Section 2.4. Briefly, 25 mg of each sample was weighed, dissolved, and subjected to an ultrasonic irradiation. The extract was then processed using Fe_3_O_4_@TEOS magnetic nanoparticles for Cd^2+^ preconcentration, followed by elution and quantification by FAAS.

All analyses were performed in triplicate, and the results were expressed as the mean concentration of Cd^2+^ in mg kg^–1^. Samples with concentrations below the LOD were reported as not detected (ND). The detection frequency and concentration ranges were calculated separately for adult and children’s products to enable comparative analysis between the two categories.

### 2.9 Health Risk Assessment

The potential health risks associated with dermal exposure to Cd in the analyzed eye shadow samples were evaluated for both noncarcinogenic and carcinogenic effects. The assessment was conducted following the guidelines established by the Scientific Committee on Consumer Safety^22^ and the United States Environmental Protection Agency.^23^ The evaluation included the calculation of the systemic exposure dose (SED), hazard quotient (HQ), and lifetime cancer risk (LCR).

#### 2.9.1 Systemic Exposure Dose

The SED was calculated to estimate the daily internal dose of Cd absorbed through the skin from the use of eye shadow products. The SED foresees the concentration of chemicals that move in the human body in a variety of ways. It is assessed considering the concentration of the metal available in the test substances, the product quantity applied to the skin in a day, using rate, or application frequency, the surface area of the applied skin, and the value of the human body weight.^17^ The value of SED can be determined using eq 2:

2 $$\mathrm{SED}\left(\mathrm{mg}/\mathrm{kg}/\mathrm{day}\right)=\left(\mathrm{Cs}\times \mathrm{AA}\times \mathrm{SSA}\times F\times \mathrm{RF}\times \mathrm{BF}\times 10^{-3}\right)/\mathrm{BW}$$

In this equation, Cs expresses the concentration of Cd in the cosmetic sample (mg/kg). AA is the concentration of the product to be applied in a day (g/cm^2^), SSA is the body surface area (cm^2^), and *F* shows the frequency for product application per day. The values of these parameters are standard and established by the Scientific Committee on Consumer Safety^22^ and assumed to be 0.02 g, 24 cm^2^, and 2 per day, respectively, for eye shadow products. RF indicates the retention factor, which is taken as 1.0 for leave-on cosmetic products. BF is the bioavailability factor, representing the fraction of the substance that is systemically absorbed; a conservative value of 0.5 (50%) was used in this assessment.^24^ The factor 10^–3^ (mg/kg) is used as a unit conversion factor, while BW highlights the average weight of human body and is projected to be 60 kg for adults and 15 kg for children in this study.^22,25^

The bioaccessibility factor (BF) was set at 0.5 (50%) following the SCCS^22^ Notes of Guidance (12th revision), which recommends a 50% default value for dermal absorption in the absence of experimentally determined data (SCCS, 2023). This default value has been consistently adopted in recent cosmetic risk assessment studies evaluating heavy metals in facial cosmetics,^17,26,27^ including studies specifically addressing eye shadows.^28^

#### 2.9.2 Hazard Quotient (HQ) and Hazard Index (HI)

The hazard quotient (HQ) indicates the ratio of the SED of a test substance to the RfD of each metal. The value of HQ can be determined through eq 3

3 HQ=SED/RfD

An HQ value less than or equal to 1 indicates that the exposure is unlikely to cause adverse noncancerous health effects, and the risk is considered acceptable. Conversely, values greater than 1 suggest potential for adverse health effects, and the risk is deemed unacceptable.^25^

#### 2.9.3 Lifetime Cancer Risk

The LCR value was determined to identify the risk of lifelong cancer due to the presence of carcinogenic metals in cosmetic creams and was measured using the following eq 4:

4 LCR=SED×CSF

Where, CSF is the coefficient of the slope of carcinogenicity and the transition to cancer risk for a unit dose of a cancer-causing substance that is consumed during a typical lifespan. The slope coefficient value for Cd is 6.7 (mg/kg/day)^−1^, as reported in the literature.^25,26^ An LCR value below 1 × 10^–6^ is considered negligible, values between 1 × 10^–6^ and 1 × 10^–4^ are generally considered acceptable, and values exceeding 1 × 10^–4^ are deemed unacceptable and indicate a significant carcinogenic risk.^29^

## 3 Results and Discussion

### 3.1 Synthesis and Characterization

Magnetic nanoparticles based on Fe_3_O_4_ have a unique structure, a high surface area-to-volume ratio, and the ability to be coated with various ligands, justifying their widespread use as a core for the preparation of various magnetic materials.^30^ However, Fe_3_O_4_ has a high tendency to aggregate with other particles, resulting in a loss of the magnetic properties. One way to reduce this aggregation is by coating them with TEOS, as this improves their chemical stability and provides greater protection against oxidation and toxicity. After silica coating, the surfaces of MNPs are replete with silanol groups, which can be modified with various coupling agents to covalently bind specific bioligands to the surfaces of the magnetic nanoparticles.^31^ The synthesis of magnetic Fe_3_O_4_ nanoparticles was successfully achieved by using the coprecipitation method. After surface modification with TEOS, Fe_3_O_4_@TEOS nanoparticles were obtained, presenting a brown to black color. A purification process involving repeated washing, stirring, and magnetic separation was then performed to remove uncoated Fe_3_O_4_ nanoparticles, which exhibited higher magnetization and a faster magnetic response. The resulting material demonstrated higher purity, which was essential for achieving a reproducible adsorption performance.

To evaluate the effect of the TEOS coating on the stability of the Fe_3_O_4_ particle dispersion, a zeta potential analysis was performed. Zeta potential is the main parameter for evaluating colloidal stability, where a high absolute value (>|30| mV) indicates sufficient repulsive forces between particles to prevent aggregation, while values close to zero indicate a tendency toward coagulation and sedimentation.^32^ The results of the zeta potential measurements show that Fe_3_O_4_@TEOS has a high zeta potential value, which increases with increasing pH, reaching −57.37 mV at pH 10.0, indicating that the particles do not tend to agglomerate in liquid media, as shown in Figure S1 (in Supporting Information). Furthermore, it is observed that the higher the pH, the more negatively charged the Fe_3_O_4_@TEOS particles become; therefore, above pH 7.0, the particles tend to repel each other, favoring the process of Cd^2+^ retention by electrostatic interaction on the surface of the nanoparticles.^33^

In SEM analyses (Figure 1), Fe_3_O_4_@TEOS showed predominantly spherical morphology with an average size of 0.659 μm. The predominance of spherical particles indicates good control of the coating process with TEOS, resulting in relatively homogeneous silica shells around the magnetic cores. Confirmation of the presence of the TEOS coating on the iron oxide nanoparticles is fundamental, as the silica shell increases particle stability against aggregation and oxidation of the Fe_3_O_4_ core and preserves the magnetic behavior of the particles, a necessary condition for their application as the magnetic phase in the extraction process. Additionally, silica-coated Fe_3_O_4_ nanoparticles exhibit better colloidal dispersivity over a wide pH range, favoring their use in different complex matrices and under varying analysis conditions.^34^

**Figure 1:**
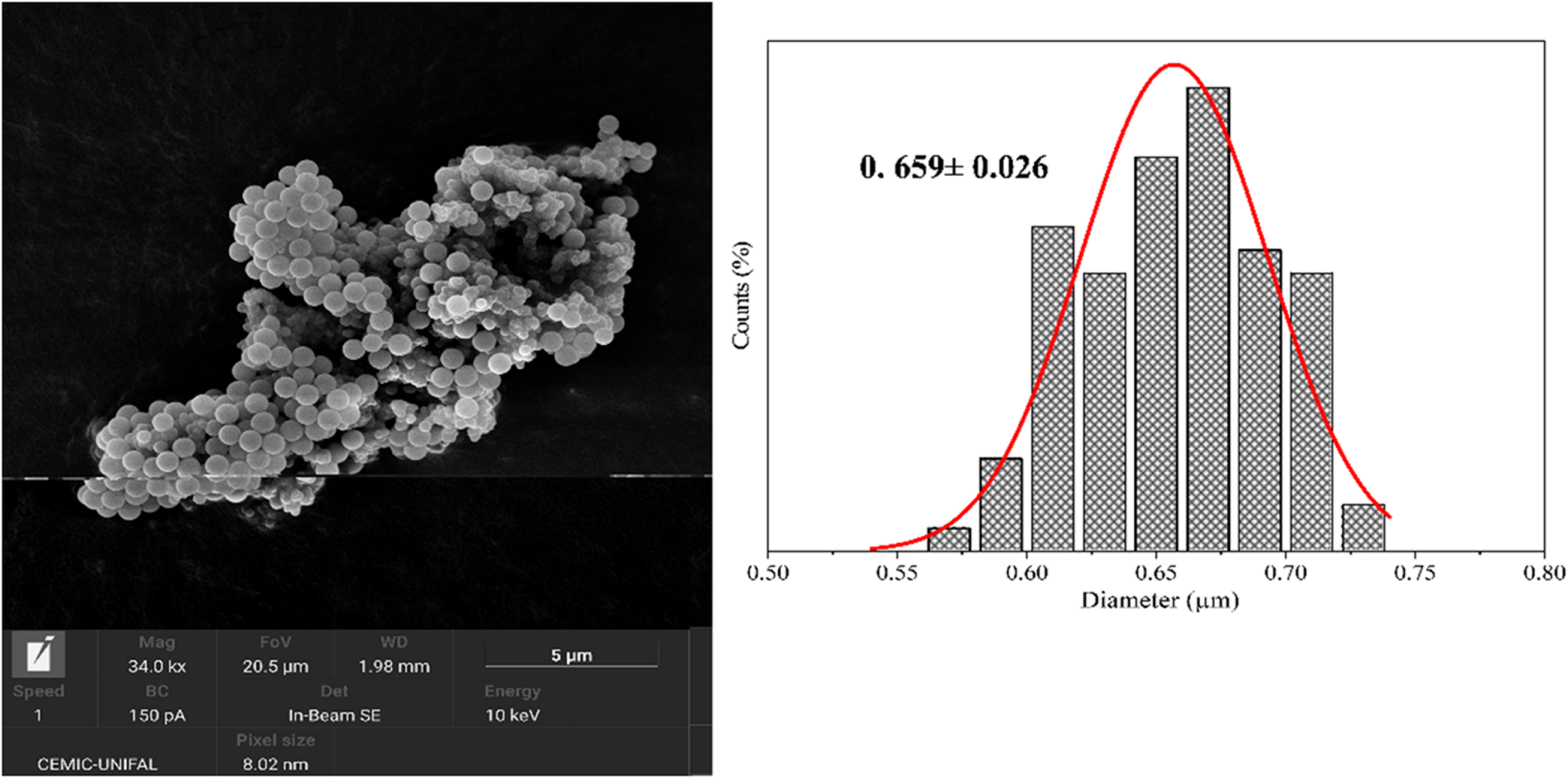
Histogram obtained from the SEM analysis for the Fe_3_O_4_@TEOS.

The X-ray diffraction pattern of Fe_3_O_4_@TEOS (Figure S2, Supporting Information) exhibited strong diffraction peaks at 2θ = 30.18, 35.62, 43.16, 57.18, and 62.84°, corresponding to the (220), (311), (400), (422), (440), and (511) planes of Fe_3_O_4_, consistent with the standard magnetite pattern (JCPDS no. 96-900-7645). These reflections confirm the face-centered cubic (fcc) crystal structure of the magnetic phase.^35^

The predominance of the (311) reflection confirms the crystalline nature of the magnetic core, in agreement with the standard ICDD/PDF sheet no. 04-0852. 96-900-7645. The absence of additional diffraction peaks suggests high purity of the material and indicates that no secondary iron oxide phase, such as hematites or maghemite, was formed during synthesis.^36^

A broad diffuse halo observed between 15 and 30° can be attributed to the amorphous silica layer formed from the hydrolysis and condensation of TEOS. Similar behavior has been widely reported for Fe_3_O_4_@SiO_2_ core–shell nanostructures, where the silica coating does not alter the crystalline structure of magnetite, but contributes to an increase in the amorphous background. Furthermore, the slight broadening of the diffraction peaks is associated with the nanometric dimensions of the particles and the possible surface disorder promoted by the silica shell.^37^

The preservation of the characteristic reflections of Fe_3_O_4_ after coating with TEOS indicates that the coating occurred predominantly on the surface of the nanoparticles, without affecting the crystalline integrity of the magnetic core. These results confirm the successful synthesis of Fe_3_O_4_-based core–shell nanoparticles coated with amorphous sílica.^36^

FT-IR spectroscopy was performed for Fe_3_O_4_@TEOS (Figure S3, Supporting Information) and revealed structural modifications on the nanoparticle surface while preserving the integrity of the magnetic core after TEOS coating. The FTIR spectrum confirmed the successful functionalization of Fe_3_O_4_ nanoparticles with silica derived from TEOS hydrolysis. A band was observed at approximately 3400 cm^–1^, attributed to O–H stretching vibrations related to residual synthesis water.^38^

Additional peaks at approximately 800, 950, and 1090 cm^–1^ corresponded to Si–O symmetric stretching, Si–OH asymmetric stretching, and Si–O–Si vibrations, respectively, and these values are consistent with the literature.^18,38,39^ These spectral features validated the successful coating of Fe_3_O_4_ with silica, which was crucial for improving the stability and adsorption properties of the nanoparticles. The Fe–O stretching vibration characteristic of magnetite was identified by a band around 580 cm^–1^, confirming the preservation of the Fe_3_O_4_ magnetic core after functionalization.^40^

### 3.2 Optimization of the Extraction Conditions

To optimize the extraction process, a 2^6–1^ fractional factorial design with triplicate of the center point was performed. To determine the appropriate extraction conditions, the following variables were evaluated: (V_1_) Fe_3_O_4_@TEOS mass (mg); (V_2_) pH; (V_3_) eluent concentration (mol L^–1^); (V_4_) adsorption time (min); (V_5_) ultrasound time (min); and (V_6_) desorption time (min). Table 1 presents the main factors: low level (−), central point (0), and high level (+), and the results obtained from the 2^6–1^ fractional factorial design. The significance of the factors and their interactions was evaluated based on absorbance (peak height). Each analytical response was examined using analysis of variance (ANOVA) and represented by a Pareto chart, with 95.0% confidence intervals. The vertical line in the chart indicates the statistical significance threshold for *p* = 0.05, as shown in Figure 2.

**Figure 2:**
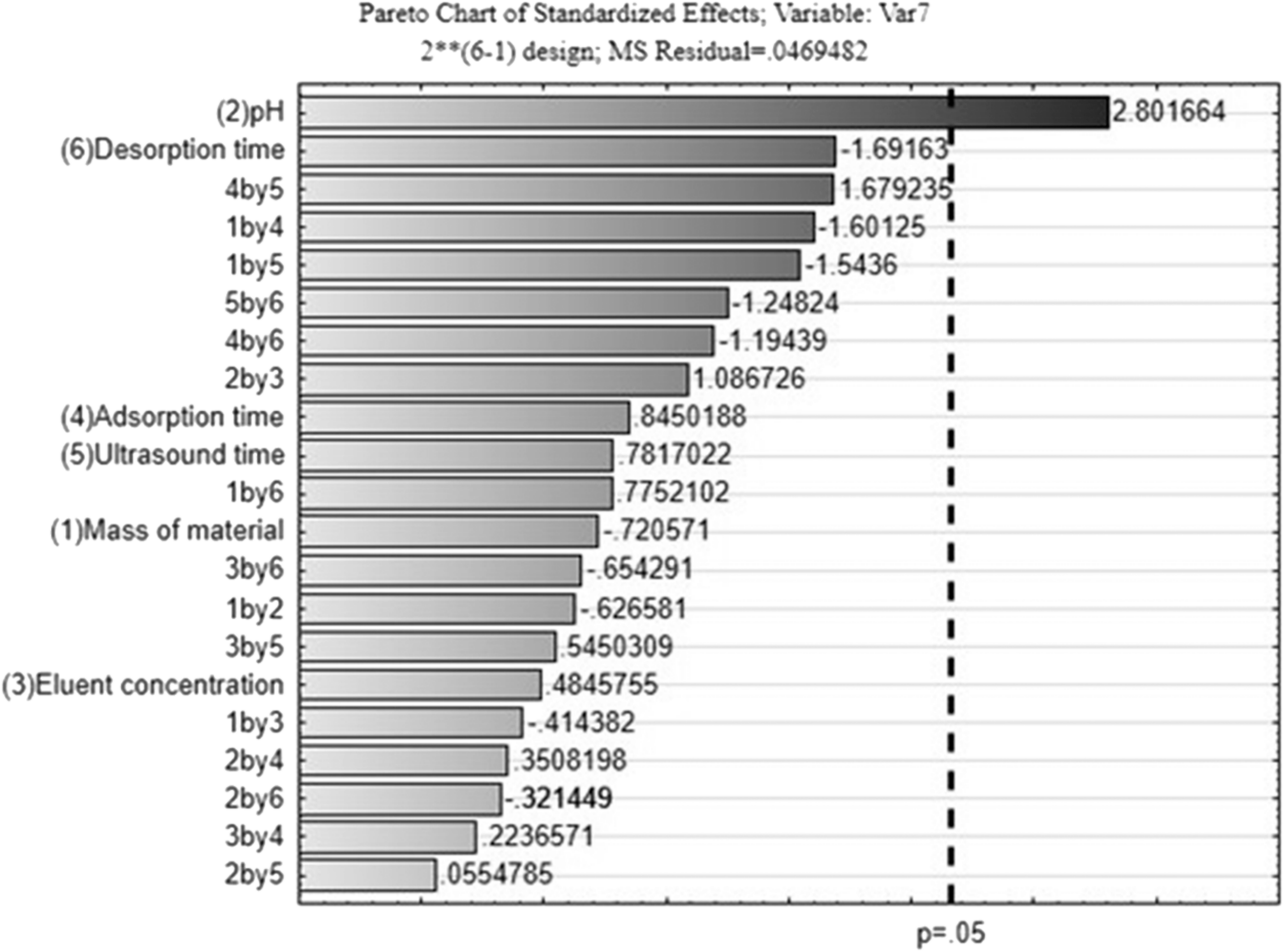
Pareto chart for the optimization of extraction conditions.

According to the Pareto chart, the only significant variable was pH, which showed a positive contrast (2.801), indicating that an increase in pH leads to an increase in the analytical response. However, it is known that different Cd species are present depending on the pH. Below pH 8.0, Cd exists almost entirely as the free Cd^2+^ ion, which is highly soluble and bioavailable. As the pH increases, hydrolysis gradually forms species such as Cd­(OH)^+^, Cd­(OH)_2_, Cd­(OH)_3_^–^, and Cd_4_(OH)_4_^2+^. Above pH 10, hydroxylated species predominate, and precipitation of Cd­(OH)_2_ may occur.^41^ Therefore, a univariate study was conducted in the pH range of 8 to 10, aiming to optimize pH to achieve more efficient extraction while preventing Cd precipitation.

The other variables investigated did not show statistical significance at the 95.0% level. Therefore, low levels were chosen for eluent concentration, adsorption time, ultrasound time, and desorption time. This choice reduces experimental time and helps minimize waste generation, in accordance with the principles of green chemistry. For the Fe_3_O_4_@TEOS mass variable, a high level was selected because, despite not being statistically significant, it provided a greater analytical response. Thus, after method optimization, the experimental conditions considered ideal for these variables were: Fe_3_O_4_@TEOS mass of 20.00 mg; eluent concentration of 0.100 mol L^–1^; adsorption time of 5.0 min; ultrasonication time of 1.0 min; and desorption time of 1.0 min.

After conducting the 2^6–1^ fractional factorial design, pH was identified as the only statistically significant variable with a positive effect on the analytical response. Therefore, a subsequent univariate study in the pH range 8.0–10.0 (ΔpH = 0.5) presented in Figure 3 was performed as a confirmatory step, using the other factors fixed at the levels selected from the factorial design.

**Figure 3:**
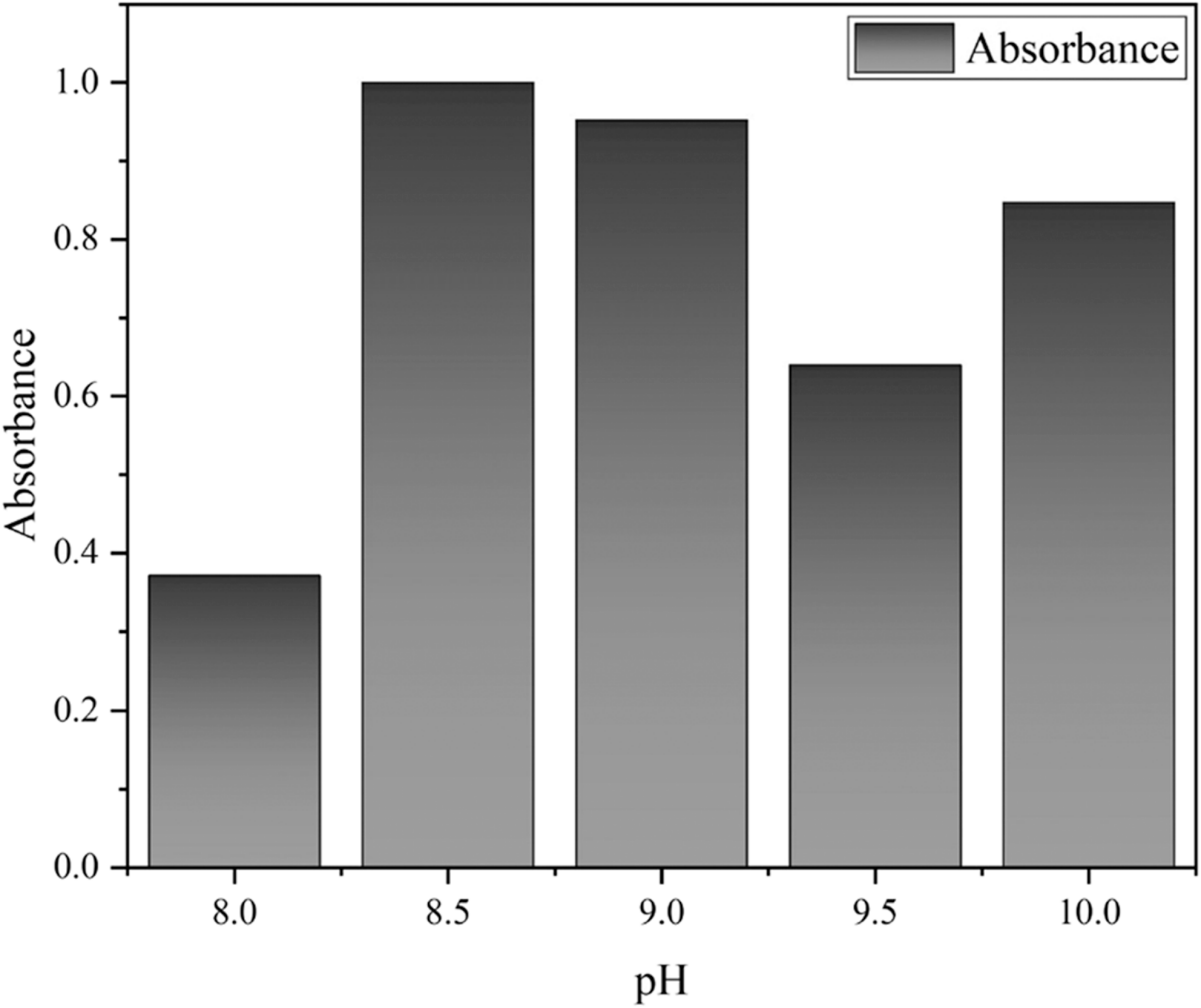
Graph of the univariate analysis of pH for Cd^2+^ extraction by Fe_3_O_4_@TEOS.

The surface charge behavior of Fe_3_O_4_@TEOS was previously evaluated by zeta potential measurements between pH 2.0 and 10.0 (Figure S1, in Supporting Information). The material reached the isoelectric point (IP) approximately at pH 2.8, after which the negative charge increased progressively with pH, reaching −57.37 mV at pH 10.0. Thus, at the working pH of 8.5, the sorbent surface is well above its IP and predominantly negatively charged, favoring electrostatic attraction with Cd^2+^ and increasing adsorption efficiency.

In the pH study in the range 8.0–10.0, signal increased up to pH 8.5, followed by loss of sensitivity at pH 9.0, 9.5, and 10.0, attributed to Cd­(OH)_2_ precipitation, as previously reported for Cd speciation at high pH.^33^ Thus, working at pH above the IP ensures a negatively charged surface, but at very basic pH values, Cd precipitation begins. Therefore, pH 8.5 was selected because it maximizes Cd^2+^ adsorption by electrostatic interaction and avoids precipitation effects.^42^

It is important to note that, considering the trace concentration levels of Cd in cosmetic samples and the previously reported adsorption capacity of the Fe_3_O_4_@TEOS material (50 mg g^–1^) by our research group in a previous study, saturation of the sorbent was not expected under the proposed extraction conditions.^38^

### 3.3 Analytical Performance

Analytical performance was evaluated based on linearity, intraday and interday precision, intraday and interday accuracy, limit of detection (LOD), and limit of quantification (LOQ). The proposed method exhibited a linear relationship from 0.010 to 0.100 mg L^–1^, with a coefficient of determination (*R* ^2^) of 0.9959 and the linear regression equation ABS = (0.0014 ± 0.00025) [Cd^2+^] + (0.0098 ± 0.0026).

Linear regression analysis was performed using OriginPro 2018 64-bit version, and the model showed excellent correlation (Pearson’s *r* = 0.99797), with an adjusted *R* ^2^ of 0.9959, confirming the goodness of fit. Analysis of variance (ANOVA) revealed a significant regression (*F* = 983.36, *p* < 0.0001), indicating a robust description of the relationship between concentration and instrumental response. These statistical parameters demonstrate the reliability of the calibration curve for quantification in the evaluated range.^43^

Intraday and interday precision and accuracy were considered adequate. The coefficients of variation ranged from 6.26 to 9.57% and 2.67 to 13.86% for intra- and interday precision, respectively. Recovery rates were 90.71 to 112.65% and 90.71 to 109.61% for intra- and interday accuracy, respectively.

There are several approaches to calculating the LOD and LOQ; in this study, they were determined in accordance with the recommendations of the International Union of Pure and Applied Chemistry (IUPAC) and calculated based on the standard deviation associated with noise obtained from ten blank measurements, and the slope of the calibration curve. This was achieved by progressively decreasing the analyte concentration in the matrix until the limits of detection and quantification were reached. As a result, the LOD and LOQ were established at 0.0016 and 0.0053 mg L^–1^, respectively. Table 2 summarizes the main results obtained in the analytical performance evaluation.

**Table 2:** Analytical Performance

Linear range (mg·L^–1^)	0.01–0.100		
Limit of detection (mg·L^–1^)	0.0016		
Limit of quantification (mg·L^–1^)	0.0053		
Intra-assay precision (CV%, *n* = 6)	2.83^a^	6.99^b^	7.88^c^
Intra-assay precision (CV%, *n* = 6, 3 days)	8.35^a^	6.62^b^	4.45^c^
Accuracy (recovery, *n* = 6)	90.71^a^	112.65^b^	104.43^c^
Accuracy (recovery, *n* = 6, 3 days)	97.38^a^	103.02^b^	107.73^c^

a 0.010 mg L^–1^ (Low concentration).

b 0.035 mg L^–1^ (Medium concentration).

c 0.100 mg L^–1^ (High concentration).

### 3.4 Comparison of the Proposed Method with Previously Reported Methods

The analytical performance of the MDSPE-FAAS method was compared with previously published procedures for Cd determination in cosmetic matrices (Table 3). Parameters such as sample, sample preparation, analytical technique, analytes, linear range, LOD, and LOQ were compared. The proposed method presents a linear range of 0.010 to 0.100 mg L^–1^ with *R* ^2^ = 0.9959 and experimentally established LOD and LOQ of 0.0016 and 0.0053 mg L^–1^, respectively. These values are of the same order of magnitude as those reported for Cd in cosmetics by other FAAS and AAS based methods, which typically operate in the range of 0.01–0.20 mg L^–1^ and report LODs around 0.001–0.01 mg L^–1^ for Cd.

**Table 3:** Comparison of the Proposed Method with Previously Reported Methods^a^

Sample	Sample preparation	Detection	Analytes	Linear range (mg L^–1^)	LOD (mg L^–1^)	LOQ (mg L^–1^)	Reference
Eye pencils, eye liners and mascara; lipsticks and lips gloss.	Conventional acid digestion	FAAS	Cd, Cr, Fe, Ni, Pb, Zn	-	-	-	^44^
Foundation, lipstick and eyeliner	Conventional acid digestion	FAAS	Fe, Cu, Zn, Pb, Ni, Cd	-	-	0.06 (Fe)	^45^
						0.03(Cu)	
						0.01 (Zn)	
						0.10 (Pb)	
						0.09 (Ni)	
						0.02 (Cd)	
Mascara and Eye Shade	Conventional acid digestion	ICP-MS	Ag, Al, As, B, Ba, Ca, Cd, Co, Cr, Cu, Fe, Hg, K, Li, Mg, Mn, Mo, Na, Ni, Pb, Rb, Sb, Se, Sn, Sr, U, V, Zn	-	-	-	^46^
Eye shadow	Microwave-assisted acid digestion	F-AAS and ICP-MS	Pb, Cd, Co, Cr, Ni	-	-	-	^47^
Body creams and lotions, powders, soaps, eye makeup products, and lipsticks widely	Conventional acid digestion	AAS	Ni, Pb, Cd, Hg, Cr	Ni and Pb (0.03–44.0); Cd (0.01–0.2); Hg and Cr (0.03–0.4)	0.005	-	^48^
Eye shadows, blushes, lipsticks, three types of lotions, mascaras, foundations, body powders, compact powders, shaving creams, and face paints	Conventional acid digestion	CVG-AFS and ICP-MS	As, Cd, Co, Cr, Hg, Ni, Pb	-	0.048 (AS)	-	^49^
					0.018 (Cd)		
					0.052 (Cr)		
					0.0066 (Co)		
					0.0010 (Hg)		
					0.032 (Ni)		
					0.0084 (Pb)		
Lipsticks, lip glosses and balms, eye shadows, eye pencils, eyeliners, mascaras, blushes and face powders	Conventional acid digestion	FAAS	Cd, Pb, Ni, Cr, Cu, Co, Fe, Mn, Zn	-	-	-	^50^
Eyeliner, eye pencil, mascara, lipstick, powder, face cream, body cream, sun block, Vaseline, and the traditional eye cosmetic (kohl).	Conventional acid digestion	FAAS	Cd, Cr, Cu, Ni, Pb	0.0005–0.005	-	-	^51^
Sweat extract of branded and nonbranded facial cosmetic products.	Conventional acid digestion	FAAS	Cd, Pb	0.001–0.010	3.70 × 10^–5^ (Cd)	-	^52^
					9.00 × 10^–5^ (Pb)		
Lipstick and eye shadow	Conventional acid digestion	ICP-MS	Cr, Co, Ni, Cu, As, Cd, Pb, Hg	-	0.0020 (Cr)	-	^53^
					0.0008 (Co)		
					0.0020 (Ni)		
					0.0020 (Cu)		
					0.0070 (As)		
					0.0040 (Cd)		
					0.0006 (Pb)		
					0.0040 (Hg)		
Eye shadow and blushes	DMSPE–FAAS	FAAS	Cd	0.010–0.1	0.0016	0.0053	This study

a TIL-DμE-ISAYS: Tandem ionic liquid-based dispersive microextraction method using in-syringe air-assisted vesicle system; FAAS: Atomic absorption spectrometry; ICP-MS: inductively coupled plasma–mass spectrometry; ICP-AES: Inductively coupled plasma atomic emission spectrometer; FIMS: Flow Injection Mercury System; CVG-AFS: Mercury by cold vapor atomic fluorescence spectrometry; atomic absorption spectrometry (AAS).

Although ICP-MS procedures can achieve lower LODs, they generally require conventional acid digestion, a higher instrument cost, and more complex operation. In contrast, the proposed MDSPE-FAAS protocol was validated directly on the complex makeup matrix, without microwave or conventional acid digestion, using widely available FAAS instrumentation and reduced consumption of concentrated acids, in accordance with green chemistry principles.

The linear range of 0.010–0.100 mg L^–1^ was established after optimization and validation in the presence of the cosmetic matrix and covers the concentration levels found in preconcentrated solutions obtained from real samples, which reached up to 25.72 mg kg^–1^ in solid products. Considering the mass of the sample applied and the preconcentration step, this range is suitable for routine monitoring of Cd in makeup, including products with Cd contents well below and well above current reference values. Within this range, the method showed satisfactory precision (CV ≤ 13.86%) and recoveries between 90.71 and 112.65%, meeting the validation requirements of ANVISA Resolution No. 166/2017 for quantitative methods in complex matrices. Therefore, when compared with previously reported methods, the proposed MDSPE-FAAS procedure offers a favorable balance between sensitivity, operational simplicity, matrix-adapted linear range, and environmental sustainability, making it suitable for routine monitoring of Cd in cosmetic products.

In addition to analytical performance, the environmental impact of the proposed procedure was evaluated using the Analytical Sustainability Metric Approach (AGREE).^54^ The method achieved an AGREE score of 0.68 (Figure 4), indicating a satisfactory level of compliance with the 12 principles of green analytical chemistry. The favorable score is mainly associated with the elimination of conventional acid digestion procedures, lower consumption of concentrated reagents, low waste generation, magnetic separation without centrifugation or filtration, and the use of readily available FAAS instrumentation.

**Figure 4:**
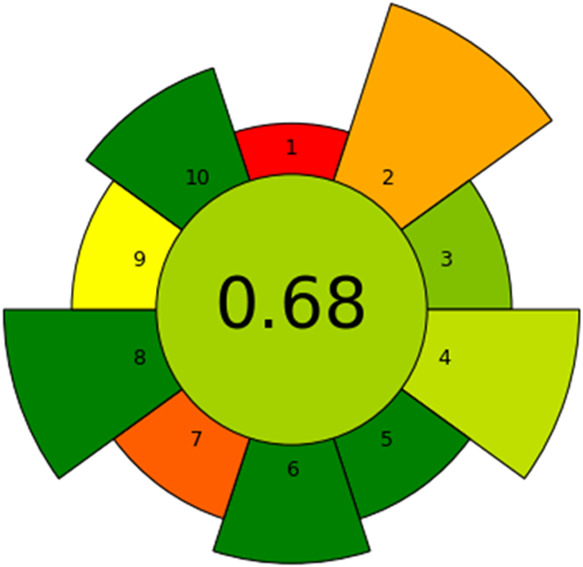
Analysis of green chemistry-related metrics.

Compared to ICP-MS-based procedures, which generally require microwave-assisted acid digestion, larger reagent volumes, and higher energy consumption, the proposed MDSPE-FAAS method offers a more sustainable and operationally simpler alternative for routine cadmium monitoring in cosmetic products. Furthermore, the direct application to complex cosmetic matrices, without the need for exhaustive sample digestion, contributes to reducing analytical costs and the environmental impact.

Therefore, the proposed method offers a suitable balance among sensitivity, precision, operational simplicity, and analytical sustainability, which justifies its applicability for the routine determination of cadmium in makeup samples.

### 3.5 Study of the Interfering Ions

The analyses were conducted as described in Section 2.7. Figure 5 shows the recovery percentages of the analytical signal for Cd^2+^ in the presence of interfering ions, such as Cu^2+^, Na^+^, K^+^, Mg^2+^, Fe^3+^, Zn^2+^, SO_4_^2–^, and HCO_3_^–^.

**Figure 5:**
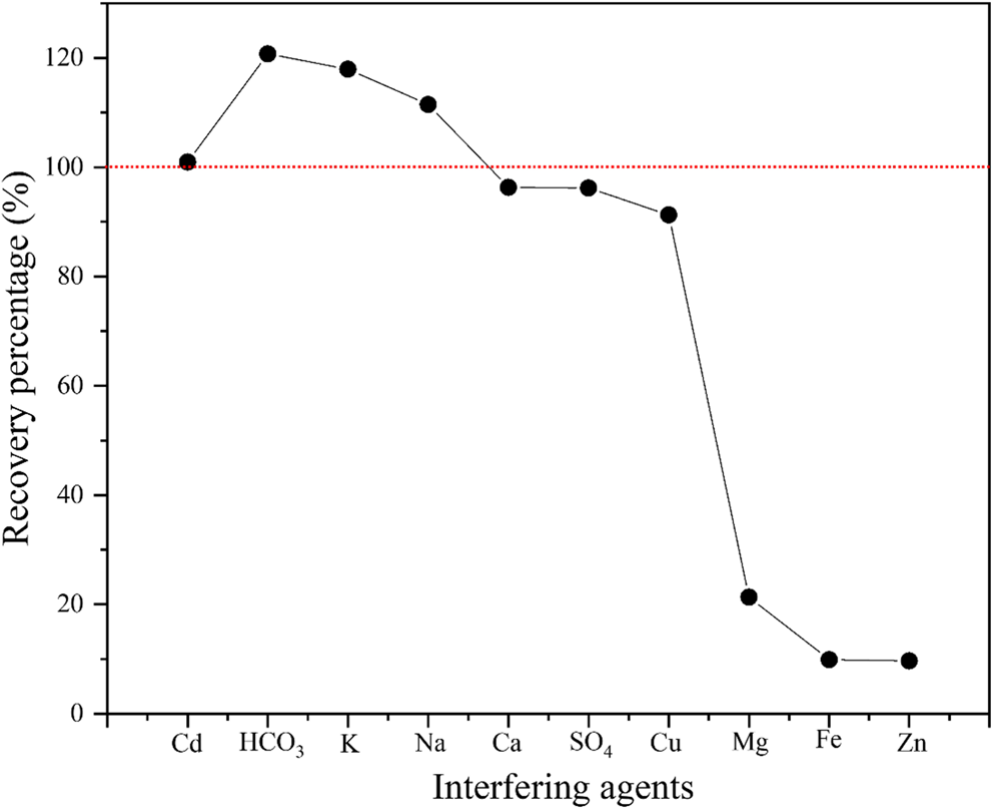
Effect of interfering ions on the recovery percentages for the Cd^2+^ analytical signal using Fe_3_O_4_@TEOS as adsorbents in the FAAS system. Conditions: Cd^2+^ (0.50 mg L^–1^); Cu^2+^, Zn^2+^, Mg^2+^, Fe^3+^, K^+^, Na^+^, Ca^2+^, SO_4_^2–^, and HCO_3_^–^ (1.00 mg L^–1^).

The results indicate that the interfering effect is most pronounced for Fe^3+^, Mg^2+^, and Zn^2+^, which exhibit a negative impact on the recovery. For Mg^2+^ and Zn^2+^, this behavior is likely due to competition for adsorption sites, as these metal ions share similar chemical properties with Cd^2+^ and may compete for active sites on the Fe_3_O_4_@TEOS material, reducing the analytical signal.^38^ The strong interference caused by Fe^3+^ is particularly relevant, as this ion can (1) form hydroxide precipitates at pH 8.5, coprecipitating Cd^2+^; (2) block the active sites of the nanoparticles through competitive adsorption; and (3) cause spectral interferences in FAAS. The literature indicates that Fe^3+^ is among the most problematic interferents in trace metal determinations by FAAS, often requiring additional separation or masking steps.^55^

### 3.6 Evaluation of the Matrix Effect

The matrix effect was evaluated by comparing the slopes of the calibration curves prepared in aqueous solvent and in the matrix extract, in accordance with guideline SANTE/11312/2021,^56^ which confirmed the suppression of the analytical signal. As illustrated in Figure 6, the calibration curves did not overlap, reinforcing the presence of a matrix effect in the proposed method for the determination of Cd in the cosmetic samples.

**Figure 6:**
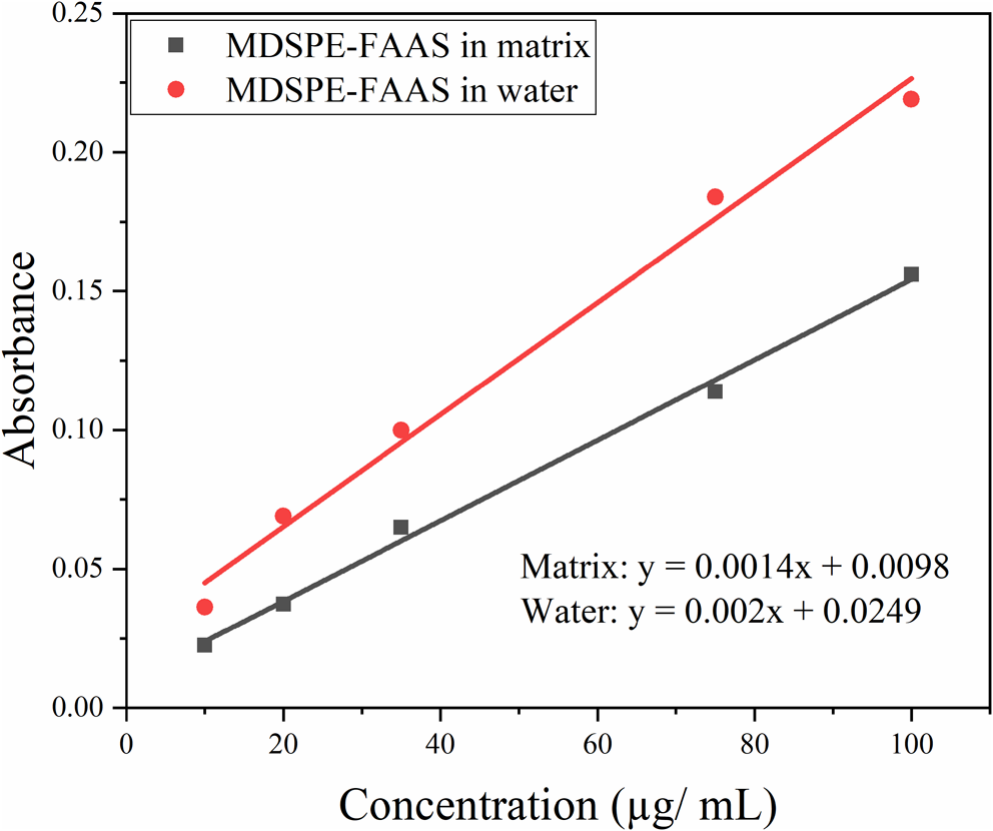
Comparison of calibration curves in solvent and matrix extract for cadmium determination.

The matrix effect was quantified using eq 2, resulting in a value of approximately 30%, indicating a moderate suppression of the analytical signal. Although individual cosmetic excipients and pigments were not separately evaluated as isolated interferents, their combined influence was inherently considered during method validation and matrix effect assessment performed directly in real cosmetic matrices with different compositions and colors. According to classification criteria reported in the literature, variations below 20% are considered insignificant, values between 20 and 50% indicate a moderate effect, while values above 50% represent a strong effect.^57^

The signal suppression can be attributed to the complexity of the makeup matrix, composed of pigments, mineral fillers, metal oxides, waxes, and oils, which can interfere with the nebulization and atomization processes during FAAS analysis.^45,58^ However, it is important to note that this effect was considered throughout the entire method validation process since all analytical parameters were established using matrix-matched calibration. Thus, although the matrix effect is present, it is effectively compensated for in the proposed method, ensuring accurate quantification of Cd in cosmetic samples. In this context, the use of the standard addition method was not considered necessary, since matrix-matched calibration proved adequate to compensate for the observed effect and ensure reliable results.^59,60^ However, for cosmetic matrices with substantially different compositions, additional calibration strategies such as the standard addition method may be required.

### 3.7 Health Risk Assessment

The health risks associated with Cd exposure from the analyzed eye shadow samples were quantified using a comprehensive risk assessment framework based on the SCCS (2023)^61^ and USEPA (2011)^25^ guidelines. The assessment evaluated the SED, HQ, and LCR for all samples containing detectable levels of Cd. Of the 29 samples analyzed, 16 (55.2%) contained detectable levels of Cd, comprising 8 adult products and 8 children’s products.

The SED values were calculated for all samples with detectable Cd concentrations using the exposure parameters established by SCCS (2023)^61^ for eye shadow products. For adult products (BW = 60 kg), the SED values ranged from 1.76 × 10^–5^ to 2.06 × 10^–4^ mg/kg/day, with a mean of 6.79 × 10^–5^ mg/kg/day. The highest SED was observed in sample 1 with a Cd concentration of 25.72 mg/kg.

For children’s products (BW = 15 kg), the SED values were considerably higher due to the lower body weight, ranging from 1.74 × 10^–5^ to 5.76 × 10^–4^ mg/kg/day, with a mean of 1.90 × 10^–4^ mg/kg/day. The highest SED was found in sample 25 with a Cd concentration of 17.99 mg/kg. These results indicate that children are exposed to approximately 2.8 times higher systemic doses compared to adults when using products with similar Cd concentrations, highlighting the increased vulnerability of this population subgroup. The data are presented in Table 4.

**Table 4:** Health Risk Assessment Parameters for Eye Shadow Samples with Detectable Cadmium Concentrations^a^

Sample	Category	Cd (mg/kg)	SED (mg/kg/d)	HQ	LCR
1	Adult	25.72	2.06 × 10^–04^	0.206	1.38 × 10^–03^
9	Adult	15.59	1.25 × 10^–04^	0.125	8.35 × 10^–04^
2	Adult	15.14	1.21 × 10^–04^	0.121	8.12 × 10^–04^
4	Adult	8.71	6.97 × 10^–05^	0.070	4.67 × 10^–04^
16	Adult	7.40	5.92 × 10^–05^	0.059	3.97 × 10^–04^
7	Adult	6.60	5.28 × 10^–05^	0.053	3.54 × 10^–04^
12	Adult	3.40	2.72 × 10^–05^	0.027	1.82 × 10^–04^
14	Adult	2.20	1.76 × 10^–05^	0.018	1.18 × 10^–04^
25	Children	17.99	5.76 × 10^–04^	0.576	3.86 × 10^–03^
21	Children	13.61	4.36 × 10^–04^	0.436	2.92 × 10^–03^
22	Children	9.25	2.96 × 10^–04^	0.296	1.98 × 10^–03^
20	Children	7.67	2.45 × 10^–04^	0.245	1.64 × 10^–03^
29	Children	5.57	1.78 × 10^–04^	0.178	1.19 × 10^–03^
24	Children	2.74	8.78 × 10^–05^	0.088	5.88 × 10^–04^
28	Children	1.96	6.26 × 10^–05^	0.063	4.20 × 10^–04^
23	Children	0.54	1.74 × 10^–05^	0.017	1.16 × 10^–04^

a HQ ≤ 1 indicate acceptable noncarcinogenic risk. LCR > 10^–4^ indicates a potential carcinogenic concern. All LCR values exceeded the acceptable threshold.

The Hazard Quotient was calculated to assess the noncarcinogenic risk associated with Cd exposure. An HQ value ≤ 1 indicates acceptable risk, while values > 1 suggest potential adverse health effects. For adult products, the HQ values ranged from 0.018 to 0.206, with a mean of 0.085. All values were well below the threshold of 1, indicating that the noncarcinogenic risk from Cd exposure through these products is acceptable.

For children’s products, the HQ values ranged from 0.017 to 0.576, with a mean of 0.237. Although higher than adult products due to the lower body weight, all HQ values remained below 1. The highest HQ (0.576) was observed in sample 25, representing approximately 58% of the acceptable threshold. These findings suggest that, from a noncarcinogenic standpoint, the daily use of these eye shadow products does not pose an immediate health risk to consumers, including children.

In contrast to the acceptable noncarcinogenic risk, the Lifetime Cancer Risk assessment revealed significant concerns. The LCR was calculated using a CSF of 6.7 (mg/kg/day)^−1^ for Cd. According to international guidelines, an LCR < 10^–6^ is considered negligible, 10^–6^ to 10^–4^ is acceptable, and >10^–4^ is unacceptable.^29^ For adult products, the LCR values ranged from 1.18 × 10^–4^ to 1.38 × 10^–3^, with a mean of 4.54 × 10^–4^. All eight samples with detectable Cd exceeded the acceptable threshold of 10^–4^, indicating a potential carcinogenic risk. The highest LCR (1.38 × 10^–3^) was observed in sample 1, corresponding to an excess cancer risk of approximately 1 in 725 individuals over a lifetime of exposure. For children’s products, the LCR values were even more concerning, ranging from 1.16 × 10^–4^ to 3.86 × 10^–3^, with a mean of 1.52 × 10^–3^. The highest LCR (3.86 × 10^–3^) was found in sample 25, corresponding to an estimated excess cancer risk of approximately 1 in 259 individuals, which is nearly 39 times higher than the acceptable limit. These findings indicate that 100% of the samples containing detectable Cd exceeded the conservative LCR threshold, suggesting a potential carcinogenic concern based on the screening model employed.

The health risk assessment revealed a striking dichotomy between noncarcinogenic and carcinogenic risks. While all samples with detectable Cd showed acceptable noncarcinogenic risk (HQ < 1), 100% of these samples exceeded the conservative threshold for carcinogenic risk (LCR > 10^–4^) based on the screening model adopted. This finding highlights the importance of conducting comprehensive risk assessments that evaluate both end points, as focusing solely on noncarcinogenic effects would have incorrectly deemed these products safe.

The apparent contradiction between acceptable noncarcinogenic risk (HQ < 1) and elevated carcinogenic risk estimates (LCR > 10^–4^) can be explained by the fundamentally different toxicological models underlying each assessment. Noncarcinogenic risk evaluation is based on a threshold model, which assumes that a safe level of exposure exists (i.e., the Reference Dose, RfD) below which adverse systemic effects are not expected to occur. In the present study, all calculated SED values remained well below the RfD of 1.0 × 10^–3^ mg/kg/day, resulting in HQ values consistently below unity. Conversely, carcinogenic risk assessment for genotoxic carcinogens such as cadmium, classified as a Group 1 carcinogen by IARC, employs a linear nonthreshold (LNT) model. This model assumes that any level of exposure carries a proportional probability of increasing cancer risk over a lifetime, with no safe threshold. Because the LNT model extrapolates risk linearly to zero dose, even minute daily dermal exposures, when integrated over a 70-year lifespan, can mathematically exceed the stringent acceptable threshold of 10^–4^. This dichotomy between noncarcinogenic and carcinogenic risk outcomes has been consistently reported in recent studies evaluating heavy metals in cosmetics.^17,26^

It should be noted, however, that the risk assessment model employed in this study involves several conservative assumptions. First, the bioaccessibility factor (BF = 0.5) was adopted following the SCCS default recommendation for substances lacking experimental dermal absorption data,^24^ consistent with the approach employed by Kicińska and Kowalczyk (2025),^27^ Irfan et al. (2022),^17^ Arshad et al. (2020),^26^ and Alvarez-Gonzales et al. (2025).^28^ Although this value is widely adopted in the cosmetic risk assessment literature, actual systemic dermal absorption of cadmium from dry powder matrices may be lower due to the limited solubility and poor skin permeation of cadmium compounds in their particulate form. Second, the CSF = 6.7 (mg/kg/day)^−1^ used in this study was derived from oral exposure data, as no dermal-specific CSF is currently available for cadmium.^62^ Although this approach is widely adopted in cosmetic risk assessment literature^15,17,26,63^ the gastrointestinal absorption kinetics and first-pass hepatic metabolism associated with oral exposure differ from dermal penetration pathways, and the direct application of an oral CSF to dermal exposure may overestimate the true carcinogenic potency. Third, the linear nonthreshold (LNT) model used for carcinogenic risk extrapolation assumes that any level of exposure carries a proportional cancer risk, which remains a subject of scientific debate for low-dose exposures. Accordingly, the LCR values reported herein should be interpreted as conservative screening estimates rather than absolute predictions of cancer incidence.

The presence of Cd at levels that indicate a potential carcinogenic risk in over half of the analyzed products, including those specifically marketed for children, represents a significant public health concern. These findings support the need for stricter regulatory limits on Cd contamination in cosmetic products and enhanced quality-control measures by manufacturers. Furthermore, consumer awareness of the potential risks associated with heavy metal contamination in cosmetics should be promoted to enable informed purchasing decisions.

The risk assessment results obtained in this study are consistent with the broader literature on heavy metals in cosmetics. Irfan et al. (2022)^17^ evaluated cadmium in fairness creams from Pakistan and reported HQ values below unity alongside total LCR values exceeding the acceptable threshold, confirming the same dichotomy between noncarcinogenic and carcinogenic risk observed herein. Arshad et al. (2020)^26^ reported HQ values up to 8.82 for Cd in lotions and sunblock creams from Pakistan, with LCR values also surpassing the permissible limit, suggesting that certain cosmetic matrices with higher skin contact area and application frequency may pose even greater risks than eye shadows. Mercan et al. (2023)^64^ also reported HQ values of 0.060–0.193 for Cd in eye shadows from Turkey, corroborating the acceptable noncarcinogenic risk observed in the present study.

From a regulatory perspective, Brazil currently lacks specific maximum limits for cadmium in finished cosmetic products. The Mercosur GMC Resolution 16/12, internalized in Brazil through ANVISA RDC 628/2022,^65^ establishes maximum metal impurity limits for organic synthetic colorants (100 mg/kg for other heavy metals including Cd), but this threshold applies exclusively to the colorant raw material and not to the finished product. When compared with international regulatory limits for finished cosmetics, the Cd concentrations found in the present study (0.54–25.72 mg kg^–1^) are of significant concern: 100% of the samples with detectable Cd exceeded the German BVL technically avoidable limit of 0.1 mg kg^–1^ (BVL, 2017), 75% exceeded the Health Canada guidance limit of 3 mg kg^–1^,^66^ and 68.8% exceeded the ASEAN guideline of 5 mg kg^–1^.^67^ These findings highlight a substantial regulatory gap in Brazil, where no specific limits for cadmium in finished cosmetic products have been established, and underscore the need for regulatory action to protect consumers, particularly children.

## 4 Conclusion

An analytical method based on MDSPE using Fe_3_O_4_@TEOS nanoparticles coupled with FAAS was successfully developed and validated for Cd determination in makeup products. The method demonstrated adequate linearity, precision, and sensitivity for the intended application. Analysis of 29 commercial samples revealed detectable Cd in 55.2% of the products, with concentrations ranging from 0.54 to 25.72 mg kg^–1^. Children’s products exhibited a higher detection frequency (61.5%) compared to adult products (50.0%).

Health risk assessment was conducted using the SED, HQ, and LCR as indicators, following SCCS (2023)^24^ and USEPA (2011)^25^ guidelines. The noncarcinogenic risk assessment demonstrated that all samples with detectable Cd presented acceptable risk levels, with HQ values ranging from 0.017 to 0.576 (all below 1). However, the carcinogenic risk assessment indicated a potential carcinogenic concern based on conservative risk modeling. All 16 samples with detectable Cd exceeded the acceptable LCR threshold of 10^–4^, with values ranging from 1.16 × 10^–4^ to 3.86 × 10^–3^. Children’s products showed higher risk values compared to adult products, with mean LCR 3.4 times higher, primarily due to the lower body weight used in calculations. The highest carcinogenic risk was observed in a children’s product, with an LCR nearly 39 times higher than the acceptable limit.

These findings highlight the need for continuous monitoring of toxic metals in cosmetics, particularly in products intended for children, and support the implementation of stricter regulatory limits on Cd contamination. The developed method demonstrates its applicability for routine quality control in the cosmetics industry.

This study has some limitations that should be acknowledged. The health risk assessment employed the SCCS-recommended default bioaccessibility factor of 0.5 (50%) due to the lack of experimentally determined dermal absorption data for cadmium in dry powder cosmetic matrices.^24^ Although this assumption is consistent with previous studies on heavy metal risk assessment in cosmetics,^17,26–28^ the actual dermal absorption of Cd from eye shadow formulations may be lower. In addition, the cancer slope factor (CSF = 6.7 (mg/kg/day)^−1^) used in this study was derived from oral exposure data, since dermal-specific CSF values for cadmium are not currently available.^62^ Therefore, the reported LCR values should be interpreted as conservative screening estimates rather than absolute risk predictions.

From an analytical perspective, the proposed method is more suitable for matrices with characteristics similar to those of the matrices evaluated in this study. For samples with significantly different compositions, the standard addition method may be more appropriate for minimizing matrix effects. Furthermore, the present work focused exclusively on Cd determination, and the applicability of the method to other toxic metals has not yet been investigated.

Future studies should include in vivo or ex vivo dermal absorption experiments using cosmetic matrices to obtain more realistic Cd bioavailability data as well as the development of dermal-specific cancer slope factors for heavy metals commonly found in cosmetics. In addition, expanding the number of analyzed samples and including products from different regions of Brazil would provide a broader assessment of Cd contamination in the Brazilian cosmetic market. Future work should also evaluate the applicability of the proposed methodology to multielemental analysis.

## Supplementary Material



## Notes

Characteristics obtained during the material characterization stage, the data were derived from the zeta potential versus pH curve, the X-ray diffractogram, and the infrared spectrum (PDF)
