# Supplementary material for: Health Risk Assessment of Cadmium in Brazilian Makeup Products Using a Novel MDSPE-FAAS Method
Source: ACS Omega. 2026 Jul 29;11(31):46568–82. doi: 10.1021/acsomega.6c04274 (PMC13470810; doi:10.1021/acsomega.6c04274)
Supplement: Supplementary file 1 [file ao6c04274_si_001.pdf]

Health Risk Assessment of Cadmium in Brazilian Makeup Products Using a Novel MDSPE-FAAS Method

Milena Secco de Souza[a†], Cristiane dos Reis Feliciano [a†], Saulo Alves de Souza[a], Grazielle Cabral de Lima[a], Nathália Carvalho Costa[a], Gabriel Oliveira Araújo Costa[a], Bruno Alves Rocha[a], Fernando Barbosa Jr.[b], Mariane Gonçalves Santos[a*]

[a]*Instrumental Analytical Chemistry Research Group – GPQAI, Institute of Chemistry, Federal University of Alfenas - Unifal-MG, Alfenas, Minas Gerais, 37130-000, Brazil.*
[b]*Analytical and System Toxicology Laboratory, Department of Clinical Analyses, Toxicology and Food Sciences, School of Pharmaceutical Sciences of Ribeirao Preto, University of Sao Paulo, Ribeirão Preto, São Paulo, 14040-903, Brazil.*

*Address correspondence to Mariane Gonçalves Santos, Instrumental Analytical Chemistry Research Group, Institute of Chemistry, Federal University of Alfenas, Alfenas, Minas Gerais, 37130-000, Brazil. E-mail: mariane.goncalves@unifal-mg.edu.br.
[†]These authors contributed equally to this work.

Syntheses and characterizations of Fe_3O_4@TEOS

The result obtained from the zeta potential analysis of the material is shown in Figure S1.

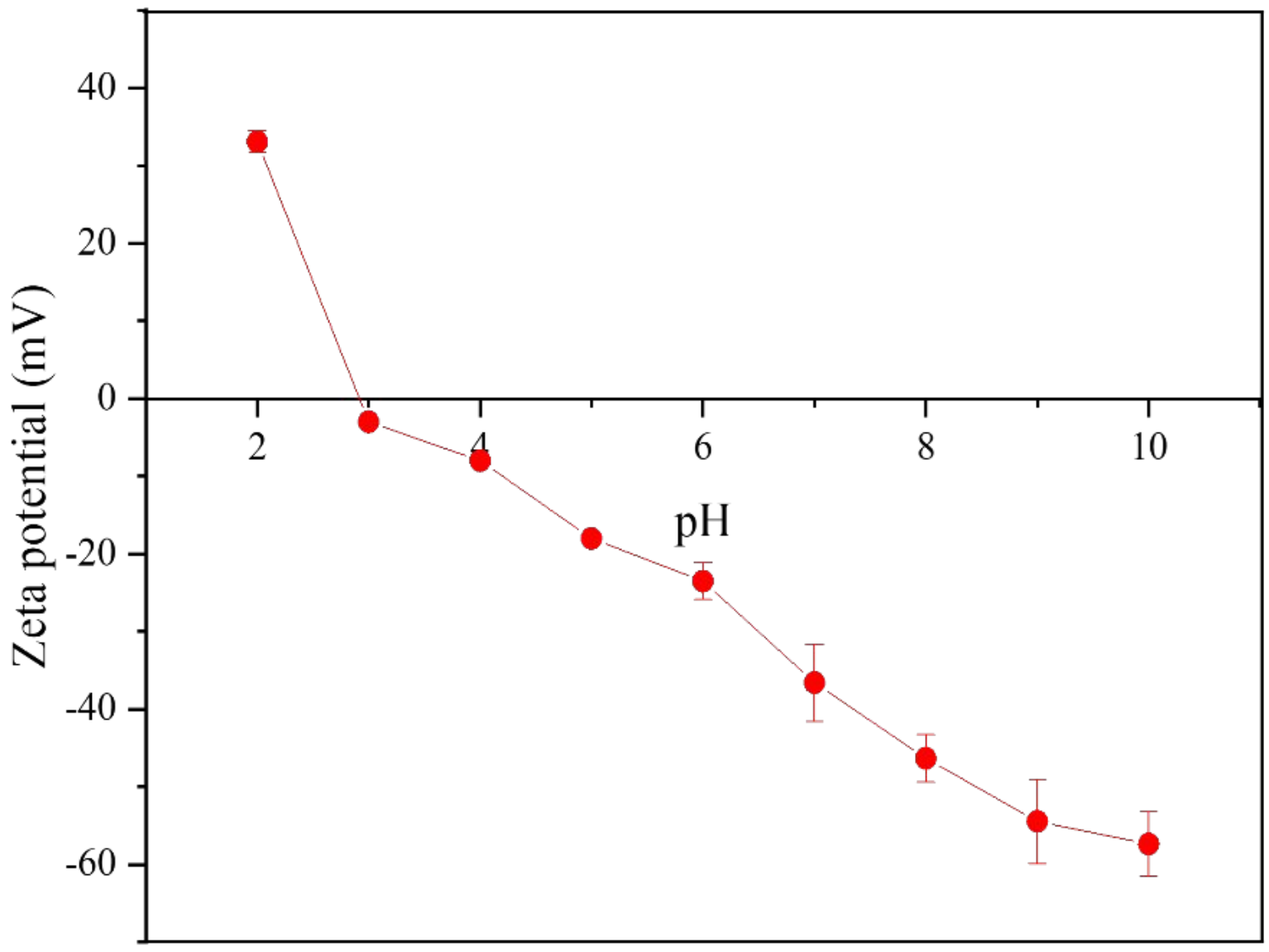

Figure S1. Zeta potential measurement of Fe_3O_4@TEOS nanoparticles as a function of pH.

An X-ray diffraction (XRD) analysis was performed, as shown in Figure S2, to investigate the crystallinity and crystal phases of the as-synthesised iron oxide nanoparticles.

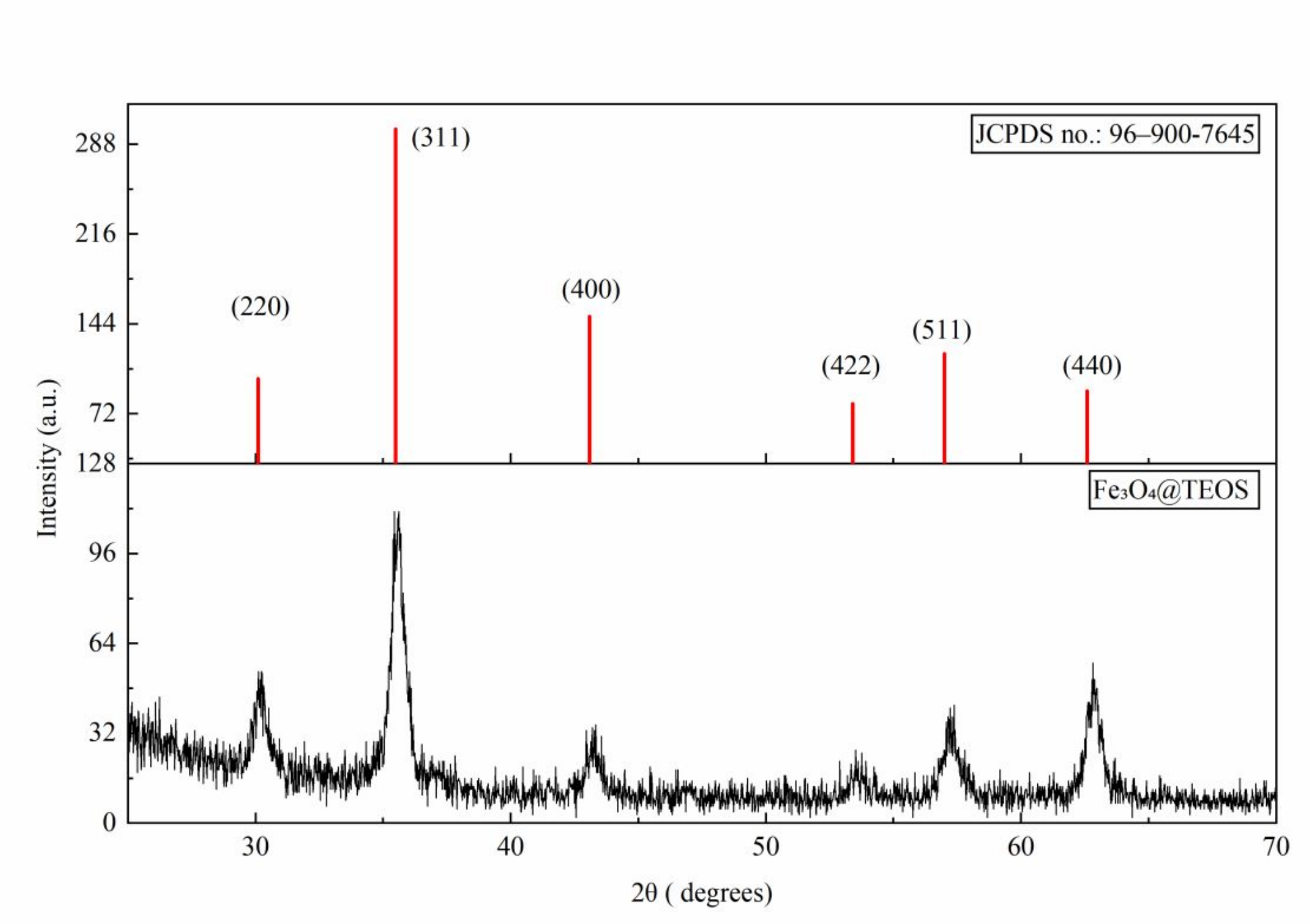

Figure S2. X-ray diffraction patterns of Fe_3O_4@TEOS nanoparticles and the corresponding standard diffraction peaks of magnetite (Fe_3O_4) indexed according to the ICDD/PDF card no. 96-900-7645

Figure S3 shows the FT-IR performed for Fe_3O_4@TEOS.

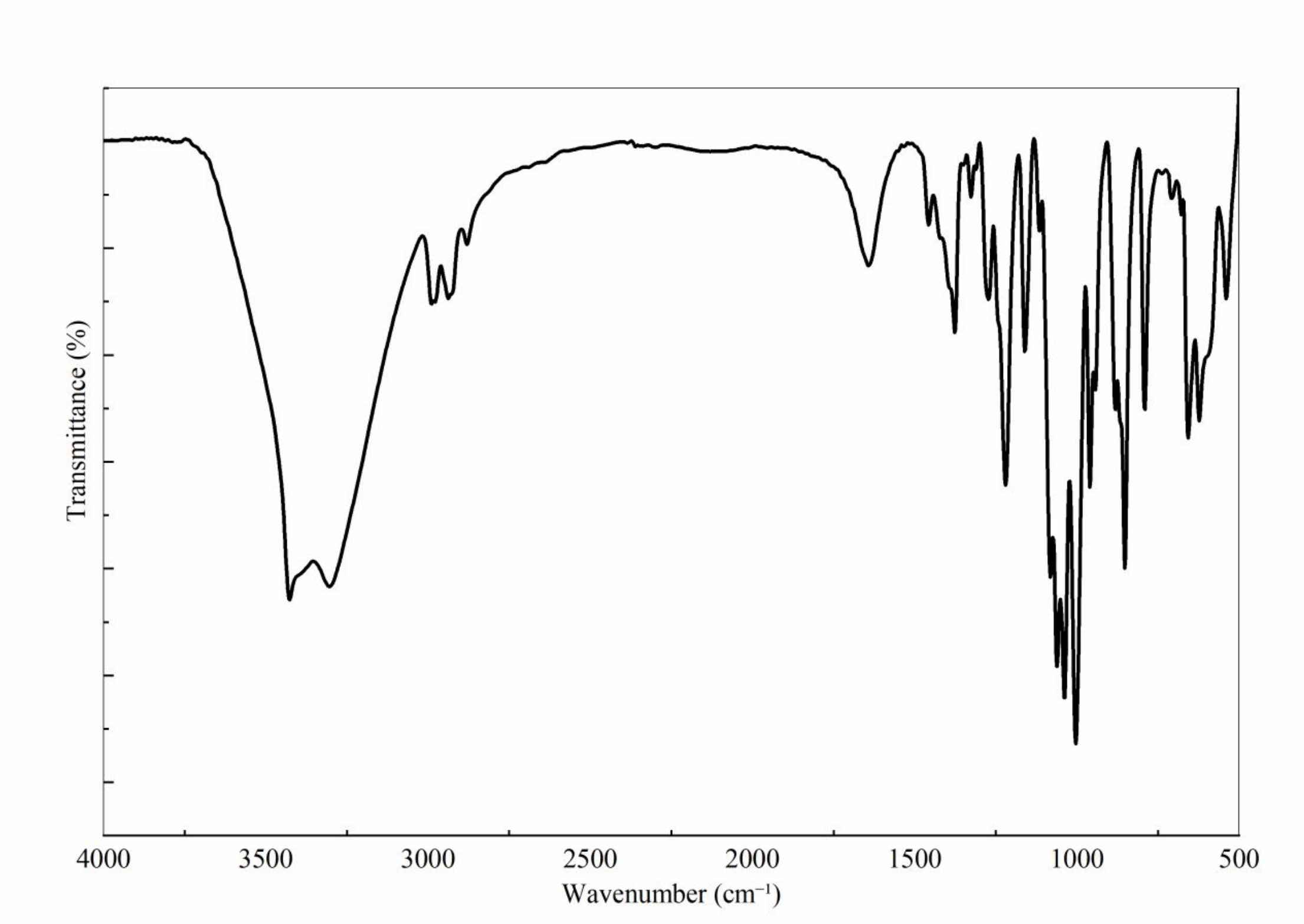

Figure S3: Fourier-transform infrared spectra of Fe_3O_4@TEOS nanoparticles.
